# Supplementary material for: Unveiling the Bioactive Potential of the Invasive Jellyfish Phyllorhiza punctata Through Integrative Transcriptomic and Proteomic Analyses
Source: Biomolecules. 2025 Aug 4;15(8):1121. doi: 10.3390/biom15081121 (PMC12383608; doi:10.3390/biom15081121)
Supplement: Supplementary file 1 [file biomolecules-15-01121-s001.zip › Suplementary Material/FileS3/PEPFOLD4/XP_065052395_5101/clusters.html]

 PEP-FOLD clusters


Download archive of all models  here   

|  |  |  |
| --- | --- | --- |
| # | file | sOPEP |
| # cluster 1 - Size: 28 |  |  |
|  | XP\_065052395\_5101-model1 | -13.4948 |
|  | XP\_065052395\_5101-model1.1 | -13.4948 |
|  | XP\_065052395\_5101-model1.2 | -13.4948 |
|  | XP\_065052395\_5101-model1.3 | -13.4948 |
|  | XP\_065052395\_5101-model1.4 | -13.4948 |
|  | XP\_065052395\_5101-model1.5 | -13.4948 |
|  | XP\_065052395\_5101-model1.6 | -13.4948 |
|  | XP\_065052395\_5101-model1.7 | -13.4948 |
|  | XP\_065052395\_5101-model1.8 | -13.4948 |
|  | XP\_065052395\_5101-model1.9 | -13.4948 |
|  | XP\_065052395\_5101-model1.10 | -13.4948 |
|  | XP\_065052395\_5101-model1.11 | -13.4948 |
|  | XP\_065052395\_5101-model1.12 | -13.4948 |
|  | XP\_065052395\_5101-model1.13 | -13.4948 |
|  | XP\_065052395\_5101-model1.14 | -13.4948 |
|  | XP\_065052395\_5101-model1.15 | -13.4948 |
|  | XP\_065052395\_5101-model1.16 | -13.4948 |
|  | XP\_065052395\_5101-model1.17 | -13.4948 |
|  | XP\_065052395\_5101-model1.18 | -13.4948 |
|  | XP\_065052395\_5101-model1.19 | -13.4948 |
|  | XP\_065052395\_5101-model1.20 | -13.4948 |
|  | XP\_065052395\_5101-model1.21 | -13.4948 |
|  | XP\_065052395\_5101-model1.22 | -13.4948 |
|  | XP\_065052395\_5101-model1.23 | -13.4948 |
|  | XP\_065052395\_5101-model1.24 | -13.4948 |
|  | XP\_065052395\_5101-model1.25 | -13.4948 |
|  | XP\_065052395\_5101-model1.26 | -13.4948 |
|  | XP\_065052395\_5101-model1.27 | -13.4391 |
| # cluster 2 - Size: 3 |  |  |
|  | XP\_065052395\_5101-model2 | -13.4879 |
|  | XP\_065052395\_5101-model2.1 | -13.4879 |
|  | XP\_065052395\_5101-model2.2 | -13.4879 |
| # cluster 3 - Size: 31 |  |  |
|  | XP\_065052395\_5101-model3 | -13.4827 |
|  | XP\_065052395\_5101-model3.1 | -13.4827 |
|  | XP\_065052395\_5101-model3.2 | -13.4827 |
|  | XP\_065052395\_5101-model3.3 | -13.4827 |
|  | XP\_065052395\_5101-model3.4 | -13.4827 |
|  | XP\_065052395\_5101-model3.5 | -13.4827 |
|  | XP\_065052395\_5101-model3.6 | -13.4827 |
|  | XP\_065052395\_5101-model3.7 | -13.4827 |
|  | XP\_065052395\_5101-model3.8 | -13.4827 |
|  | XP\_065052395\_5101-model3.9 | -13.4827 |
|  | XP\_065052395\_5101-model3.10 | -13.4827 |
|  | XP\_065052395\_5101-model3.11 | -13.4827 |
|  | XP\_065052395\_5101-model3.12 | -13.4827 |
|  | XP\_065052395\_5101-model3.13 | -13.4827 |
|  | XP\_065052395\_5101-model3.14 | -13.4827 |
|  | XP\_065052395\_5101-model3.15 | -13.4827 |
|  | XP\_065052395\_5101-model3.16 | -13.4827 |
|  | XP\_065052395\_5101-model3.17 | -13.4827 |
|  | XP\_065052395\_5101-model3.18 | -13.4827 |
|  | XP\_065052395\_5101-model3.19 | -13.4827 |
|  | XP\_065052395\_5101-model3.20 | -13.4827 |
|  | XP\_065052395\_5101-model3.21 | -13.4827 |
|  | XP\_065052395\_5101-model3.22 | -13.4827 |
|  | XP\_065052395\_5101-model3.23 | -13.4827 |
|  | XP\_065052395\_5101-model3.24 | -13.4827 |
|  | XP\_065052395\_5101-model3.25 | -13.4827 |
|  | XP\_065052395\_5101-model3.26 | -13.4827 |
|  | XP\_065052395\_5101-model3.27 | -13.4827 |
|  | XP\_065052395\_5101-model3.28 | -13.4827 |
|  | XP\_065052395\_5101-model3.29 | -13.4827 |
|  | XP\_065052395\_5101-model3.30 | -13.4827 |
| # cluster 4 - Size: 37 |  |  |
|  | XP\_065052395\_5101-model4 | -13.4779 |
|  | XP\_065052395\_5101-model4.1 | -13.4779 |
|  | XP\_065052395\_5101-model4.2 | -13.4779 |
|  | XP\_065052395\_5101-model4.3 | -13.4779 |
|  | XP\_065052395\_5101-model4.4 | -13.4779 |
|  | XP\_065052395\_5101-model4.5 | -13.4779 |
|  | XP\_065052395\_5101-model4.6 | -13.4779 |
|  | XP\_065052395\_5101-model4.7 | -13.447 |
|  | XP\_065052395\_5101-model4.8 | -13.447 |
|  | XP\_065052395\_5101-model4.9 | -13.447 |
|  | XP\_065052395\_5101-model4.10 | -13.447 |
|  | XP\_065052395\_5101-model4.11 | -13.447 |
|  | XP\_065052395\_5101-model4.12 | -13.447 |
|  | XP\_065052395\_5101-model4.13 | -13.447 |
|  | XP\_065052395\_5101-model4.14 | -13.447 |
|  | XP\_065052395\_5101-model4.15 | -13.447 |
|  | XP\_065052395\_5101-model4.16 | -13.447 |
|  | XP\_065052395\_5101-model4.17 | -13.447 |
|  | XP\_065052395\_5101-model4.18 | -13.447 |
|  | XP\_065052395\_5101-model4.19 | -13.447 |
|  | XP\_065052395\_5101-model4.20 | -13.447 |
|  | XP\_065052395\_5101-model4.21 | -13.447 |
|  | XP\_065052395\_5101-model4.22 | -13.447 |
|  | XP\_065052395\_5101-model4.23 | -13.447 |
|  | XP\_065052395\_5101-model4.24 | -13.447 |
|  | XP\_065052395\_5101-model4.25 | -13.447 |
|  | XP\_065052395\_5101-model4.26 | -13.447 |
|  | XP\_065052395\_5101-model4.27 | -13.447 |
|  | XP\_065052395\_5101-model4.28 | -13.447 |
|  | XP\_065052395\_5101-model4.29 | -13.447 |
|  | XP\_065052395\_5101-model4.30 | -13.447 |
|  | XP\_065052395\_5101-model4.31 | -13.447 |
|  | XP\_065052395\_5101-model4.32 | -13.447 |
|  | XP\_065052395\_5101-model4.33 | -13.447 |
|  | XP\_065052395\_5101-model4.34 | -13.447 |
|  | XP\_065052395\_5101-model4.35 | -13.447 |
|  | XP\_065052395\_5101-model4.36 | -13.447 |
| # cluster 5 - Size: 1 |  |  |
|  | XP\_065052395\_5101-model5 | -13.441 |
|  |  |  |
